# HLA-F and MHC-I Open Conformers Bind Natural Killer Cell Ig-Like Receptor KIR3DS1

**DOI:** 10.1371/journal.pone.0163297

**Published:** 2016-09-20

**Authors:** Aura Burian, Kevin L. Wang, Kathryn A. K. Finton, Ni Lee, Akiko Ishitani, Roland K. Strong, Daniel E. Geraghty

**Affiliations:** 1 The Clinical Research Division, Fred Hutchinson Cancer Research Center, 1100 Fairview Ave. N., Seattle, WA, 98109, United States of America; 2 The Division of Basic Sciences, Fred Hutchinson Cancer Research Center, 1100 Fairview Ave. N., Seattle, WA, 98109, United States of America; 3 Nara Medical University, Kashihara, Nara 634, Japan; Saint George's University, UNITED KINGDOM

## Abstract

Based on previous findings supporting HLA-F as a ligand for KIR3DL2 and KIR2DS4, we investigated the potential for MHC-I open conformers (OCs) as ligands for KIR3DS1 and KIR3DL1 through interactions measured by surface plasmon resonance. These measurements showed physical binding of KIR3DS1 but not KIR3DL1 with HLA-F and other MHC-I OC while also confirming the allotype specific binding of KIR3DL1 with MHC-I peptide complex. Concordant results were obtained with biochemical pull-down from cell lines and biochemical heterodimerization experiments with recombinant proteins. In addition, surface binding of HLA-F and KIR3DS1 to native and activated NK and T cells was coincident with specific expression of the putative ligand or receptor. A functional response of KIR3DS1 was indicated by increased granule exocytosis in activated cells incubated with HLA-F bound to surfaces. The data extend a model for interaction between MHC-I open conformers and activating KIR receptors expressed during an inflammatory response, potentially contributing to communication between the innate and adaptive immune response.

## Introduction

A major genetic determinant of the immune response is contained within the major histocompatibility complex (MHC) including the classical class I loci HLA-A, -B and -C, whose role in immunological recognition is now well understood [1]. In addition to their role in the adaptive immune response, MHC-I also interact with the innate immune response through polymorphic killer cell immunoglobulin-like receptors (KIR), a subset of which can distinguish different MHC allotypes [2]. Consequently, *KIR* involvement in transplantation [3], pregnancy [4] and infectious disease [5], have been examined extensively through genetic association studies.

The human MHC also contains three nonclassical class I genes (*HLA-E*, *F*, *and G*) with divergent immune function. *HLA-G*, expressed from placental trophoblast cells, may function as an important tolerogenic immunoregulator during pregnancy [6]. HLA-E complex, expressed ubiquitously in coordination with classical MHC class I, interacts with CD94 combined with distinct NKG2 subunits to inhibit and activate NK cells and subsets of T cells [7]. *HLA-F* is expressed as a protein independent of bound peptide [8] and surface expression is upregulated in conjunction with classical MHC-I upon activation of lymphocytes and monocytes [9]. Expressed during an inflammatory response as open conformers (OC), both HLA-F and MHC-I OC have been implicated in a novel pathway for uptake of extracellular antigen for cross presentation [10]. HLA-F binds most allelic forms of MHC-I OC; however, HLA-F does not bind MHC-I peptide complexes. This physical interaction likely stabilizes the otherwise unstable MHC-I OC, contributing to their facility as ligands for a specific subset of killer Ig-like receptors (KIRs) [11].

Based on recent findings of receptor/ligand interactions between KIR3DL2/KIR2DS4 and MHC-I open conformers, their potential as ligands for other KIR was tested through biophysical, biochemical, and cellular staining to identify specific interactions between KIR3DS1/L1 and MHC-I open conformers. These findings were extended with native NK and T cell lines where surface binding of HLA-F and KIR3DS1 was measured coincident with specific expression of the putative ligand or receptor. The data extend a model for a broader interaction between MHC-I open conformers–including prototype HLA-F–and KIR receptors, contributing uniquely to immune recognition during an inflammatory response.

## Results

### SPR distinguishes binding of KIR3DL1S1 to HLA-F and MHCI

For the present work, KIR3DS1 and KIR3DL1 including the complete extracellular domains, D0, D1, and D2 and a portion of the stem structure with an attached His tag at the C-terminus were produced in insect cells. As a preliminary screen for binding to MHC-I, surface plasmon resonance was used with MHC-I peptide complex (trimeric MHC/peptide/beta2-m complexes) and open conformer on the surface, and recombinant KIR3DL1 and KIR3DS1 as the mobile phase, or analyte. KIR3DL1 has a well-defined receptor-ligand relationship with MHC-I, binding specifically to MHC-I peptide complex with the Bw4 epitope, and not binding to MHC-I with the Bw6 epitope (nor to MHC-I with neither epitope) [12]. The known binding characteristics facilitated the use of KIR3DL1 as a control for binding to MHC-I peptide complex and MHC-I OC, in addition to testing the integrity of the allelic MHC-I used in the experiments.

**Fig 1:**
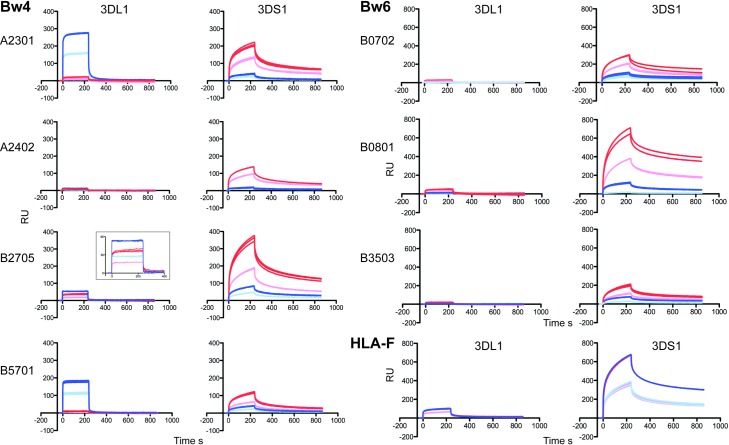
KIR3DL1 and KIR3DS1 binding to MHC-I Bw4, Bw6, and HLA-F epitopes as measured by surface plasmon resonance (SPR). Sensorgrams of KIR3DL1 and KIR3DS1 binding–indicated above each column of graphs–to immobilized refolded Bw4 epitopes of HLA heavy chain, β_2_M and respective peptide for each HLA complex as indicated. Experiments were performed in triplicate on the same chip before (blue) and after (red) acid treatment. KIR3DL1 and KIR3DS1 concentrations of 1 μM and 2 μM are indicated by light and dark colors respectively. SPR profiles for additional Bw4 and non-Bw4 allotypes, plus controls for the integrity of each complex and free heavy chain/open conformer generated after acid treatment are presented in supplementary S1 Fig and S2 Fig.

MHC-I for each of the Bw4 and non-Bw4 epitope groups were tested in SPR protocols with KIR and control analytes examining MHC-I peptide complex and MHC-I OC surfaces. These experiments demonstrated the expected specificity of the recombinant KIR3DL1 for its Bw4 ligand–and not Bw6 –as MHC-I complex and showed the absence of binding to either allotype as open conformer (Fig 1 and S1 Fig). A single Bw4 allele, HLA-A*24:02 did not bind to KIR3DL1, and this was likely due to the peptide used in refolding. Prior studies showed that specific peptides abrogated KIR3DL1 binding to MHCI complex [13, 14], and this result is reproduced by the SPR data for the A*2402 complex used here. This peptide specific binding provides additional control for the application of the SPR methodology and the biochemicals used as an effective method for testing KIR ligand interactions.

In contrast to KIR3DL1, KIR3DS1 bound both Bw4 and Bw6 MHC-I in the open conformer form but not the MHC-I peptide complex. In addition, binding to HLA-F–a stable open conformer before and after acid treatment–was higher than most of the MHC-I allotypes tested. HLA-F also showed reactivity with KIR3DL1 in this assay, although at some 8-fold lower quantitative levels. It is not clear whether these binding levels are nonspecific or reflect true physical interactions, while the binding kinetics do reflect that seen for Bw4 alleles binding to KIR3DL1. All of the complex forms were converted into open conformers using mild acid treatment [11], and controls for the integrity of both forms are presented in S2 Fig LILRB2 (ILT4) was used as a positive control for both free HCs and heterotrimeric MHCs. The high on- and off- rates of LILRB2 interaction with the free heavy chain surface born after acid treatment supports an ordered structure for MHC-I OC and argues against aggregate formation and nonspecific interaction with free heavy chains. These results are aligned with previous LILRB2 binding data from refolded HLA-G and HLA-C*04 free heavy chains [15] and also refolded HLA-F molecules [8, 11]. Additional MHC-I allotypes that lacked both the Bw4 and Bw6 epitopes were tested in SPR and neither the complex nor MHC-I OC showed significant binding to KIR3DL1 (S1 Fig). The MHC-I examined included four HLA-A, seven HLA-B, and two HLA-C alleles.

### Biochemical binding confirms the interactions between KIR3DS1 and HLA-F

**Fig 2:**
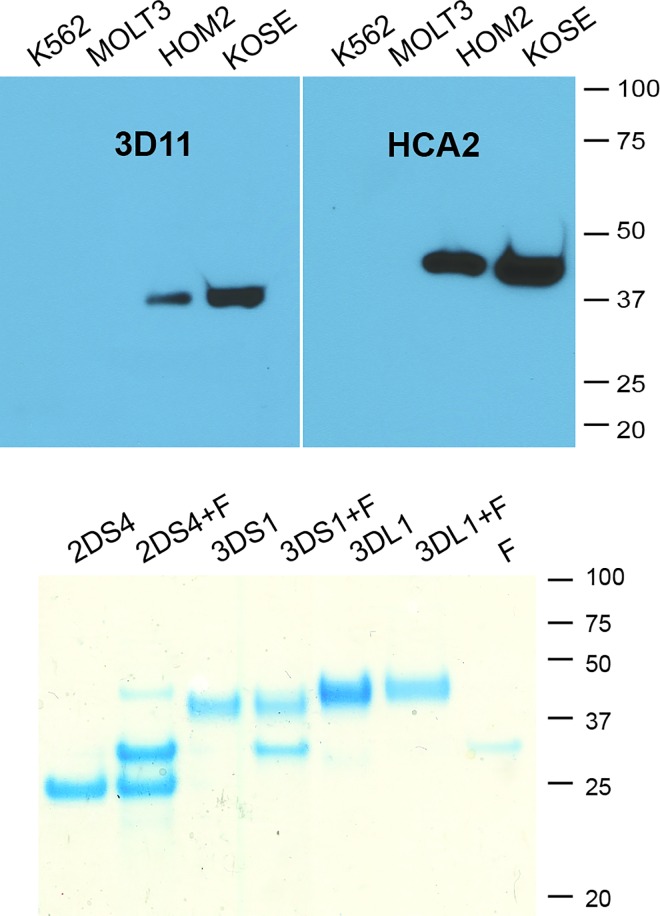
Western analysis using the indicated mAbs for detection of gel fractionated protein after pull-down with KIR3DS1-D0-D2stem-His (*above*). Four cell lines were incubated with KIR3DS1-His and pull-downs performed followed by Western blot analysis with HCA2 and 3D11. Heterodimerization of KIR-His and HLA-F without tag followed by Ni column purification (*below*). After gel fractionation, gels were stained with Coomassie blue and proteins visualized. Heterodimerization assays were carried out with KIR alone or with KIR + HLA-F as indicated above each lane. Recombinant HLA-F is included alone for comparison. MW markers are indicated for both gels.

To complement the SPR results, we performed pull-down experiments with KIR3DS1-His from cell lines expressing no MHC-I (K562), only complex form (Molt3), and two B-LCLs surface expressing both trimeric MHC-I peptide complex and MHC-I OC including HLA-F. Pull-down products were subjected to western analysis to detect HLA-F (3D11) and MHC-I (HCA2). Both proteins were detected only from the cells expressing open conformer and not from cells expressing only MHC-I complex (Fig 2 *upper*). As a third measure, heterodimerization assays were performed using KIR3DS1-His and KIR3DL1-His combined with HLA-F in comparison with KIR2DS4-His [11]. Recombinant His-tagged KIRs were individually incubated in solution with recombinant HLA-F without tag and protein was recovered from solution using Ni-NTA binding. Eluates were then fractionated on a gel and protein was observed using Coomassie blue. Consistent with previous experiments, KIR2DS4 co-eluted HLA-F in near equimolar quantities (Fig 2 *lower*). Similarly, KIR3DS1 also bound HLA-F while KIR3DL1 was eluted alone.

### HLA-F tetramer binds specifically to KIR3DS1+ NK cells

**Fig 3:**
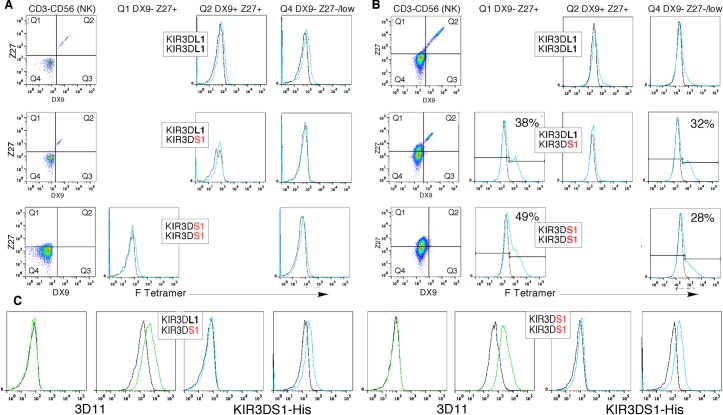
Surface staining with KIR3DS1-His and HLA-F (A) PBMC from three individuals were stained with NK markers (CD3-, CD56+) and with mAb Z27 and DX9 to detect KIR3DL1 (DX9+, Z27+) and KIR3DS1 (DX9-, Z27+), and stained with HLA-F tetramer as indicated. Three individuals were chosen based on their KIR haplotypes, containing homozygous KIR3DL1, heterozygous KIR3DL1, KIR3DS1, and homozygous KIR3DS1 as indicated. (B) Cells described in A were activated and subjected to the same staining protocols 11 days post activation. (C) Isolated T cells from two individuals with the indicated KIR3DS1L1 genotypes were stained with mAb 3D11 and with KIR3DS1-His as marked beneath each pair of before (*left*) and after (*right*) activation profiles.

HLA-F is surface expressed on activated lymphocytes and monocytes [9], and KIR3DS1 is upregulated on subsets of NK cells upon activation [16], together providing cellular targets to test for specific binding between KIR3DS1 and HLA-F. We took advantage of the genetics of KIR, where the KIR3DS1 gene is found on the KIR B haplotype while KIR3DL1 is found on the KIR A haplotype, to test for possible KIR3DS1 binding with HLA-F tetramer. Three individuals with corresponding heterozygous and homozygous KIR3DS1, KIR3DL1 genotypes were analyzed for HLA-F tetramer binding before and after activation. None of the samples expressed detectable KIR3DS1 in the resting state, and none of the resting NK cell populations showed binding to HLA-F tetramer prior to activation (Fig 3A). After activation, as expected, the KIR AA individual was negative for Z27+ DX9- cells, and all other quadrants lacked detectable binding to HLA-F tetramers. Both the KIR3DS1/L1 heterozygote and the KIR3DS1 homozygote upregulated KIR3DS1 after activation, and upregulation was coincident with binding of HLA-F tetramer to the same cell population (Fig 3B). Because of the weak reactivity of Z27 it was not possible to completely separate KIR3DS1 expressing from nonexpressing NK. Therefore, for the FACS profiles shown in Fig 3B the KIR3DS1+ cells in quadrant 1 are mixed with KIR3DS1- cells and similarly in quadrant 4, in reflected proportion. HLA-F tetramer binding showed partial staining of quadrant 1 cells with staining of quadrant 4 cells at a lower proportion, coincident with the distribution of KIR3DS1+ NK. We point out however, that the inability to cleanly separate KIR3DS1+/- cells in these experiments does leave open the possibility of HLA-F tetramer binding to other receptors in KIR3DS1+ individuals that are not present in KIR3DS1- individuals.

To examine the reciprocal KIR3DS1 binding to surface HLA-F, T cells were isolated from peripheral blood and tested before and after activation for surface staining with the recombinant KIR. FACS profiles using KIR3DS1-His showed binding consistent with HLA-F upregulation in proportion to activation levels of HLA-F for two individuals tested (Fig 3C). These correlative findings further support a receptor-ligand relationship between KIR3DS1 and MHC-I OCs.

### Functional analysis of KIR3DS1 and HLA-F interactions

**Fig 4:**
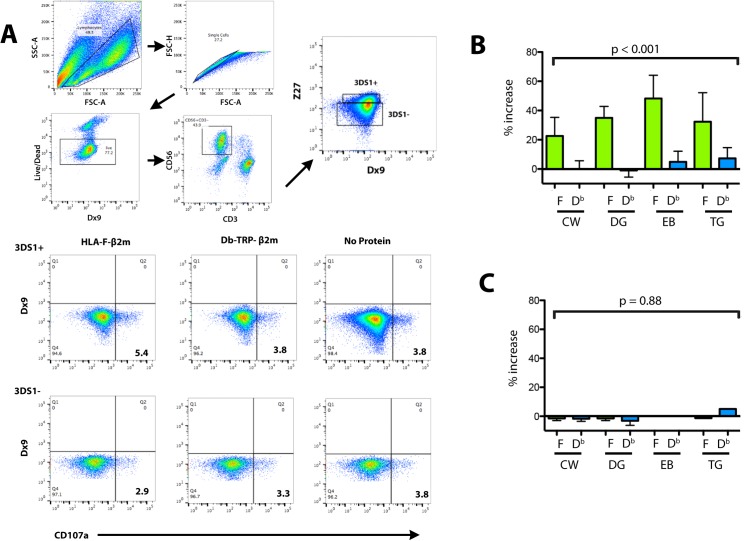
Functional measurement of HLA-F and KIR3DS1 interaction. PBMC from three donors were activated and stimulated with refolded HLA-F, HLA-control tetramers, or no protein as described in *Materials and Methods*. (**A)** A representative FACS staining for the analysis of CD107a expression on cell populations gated in the top panel indicated as KIR3DS1+ and KIR3DS1-. Expression of CD107a was detected with percentages of the total cell population analyzed in bold in the lower right quadrant of each profile. Experiments were performed in the presence of HLA-F and D^b^-TRP bound to streptavidin beads and no-protein as indicated above the panels. The percentages CD107a positive cells within the KIR3DS1+ populations (**B**) and in the KIR3DS1- population (**C**) for each donor are graphed for comparison. **(B and C)** Five experiments were performed for each of four KIR3DS1+ donors (Donors CW and EB: KIR3DL1/KIR3DS1, Donors TG and DG KIR3DS1/KIR3DS1). The graphs show the percentage of total cells using the formula $\frac{\%(F\;or\;D^{b})-\%control}{\%control}$ showing the mean of 5 replicates for each individual, the error bars represent the standard error of the mean (SEM) of the replicates, and the p-values calculated by performing a paired t-test comparing all values F and D^b^ across replicates and individuals.

A demonstration of functional interactions with KIR3DS1 has previously been limited to measurement of the effects of receptor cross-linking on granule exocytosis (CD107a expression) on NK after PBMC stimulation with mAb Z27 [17]. Experiments were designed in order to test the ability of HLA-F tetramer to act as cross-linking agent in a manner similar to that demonstrated using mAb Z27. Using KIR3DS1+ individuals stimulated for upregulation of KIR3DS1 as above, soluble HLA-F tetramers, Db tetramers as comparative control, and no tetramer as negative control, were incubated with stimulated cells as described in Materials and Methods. Expression of CD107a was measured on gated KIR3DS1+ and KIR3DS1- cells for each experimental condition demonstrating an increased CD107a expression pattern on Z27+, Dx9- cells incubated with HLA-F in three individuals (Fig 4). The increased CD107a in HLA-F tetramer incubations was specific to the KIR3DS1+ population and did not reflect an increase over background or Db tetramer incubations in CD56+, KIR3DS1- cells. These experiments were replicated a total of 3 times and the increase in HLA-F stimulated CD107a cells exceeded levels of the maximum responses seen from the HLA-control tetramer and negative control stimulations raging from 10 to 110% higher (Fig 4B and 4C).

## Discussion

Knowledge of the ligand for KIR3DS1 has thus far proven elusive. The data presented here include biophysical detection of binding using recombinant proteins and demonstrated biochemical interactions with native proteins expressed on the cell surface. Although the binding of KIR3DS1 with HLA-F expressing T cells is correlative evidence, the binding of HLA-F tetramer to KIR3DS1+ NK is not simply correlative. The KIR3DL1 homozygous individual did not show binding under activation conditions identical to those used for the KIR3DS1+ individuals who did show HLA-F tetramer binding after activation. While there are other receptors changing expression upon activation, they are changing on cells from all three genotypes similarly. In the aggregate, the biochemical, biophysical, and cell surface binding studies are consistent with a physiological role for a receptor-ligand relationship between KIR3DS1 and MHC-I open conformers, including HLA-F.

*KIR3DS1* and *KIR3DL1* are functionally divergent alleles, and not distinct loci, with divergent signaling domains and highly similar external domains [18]. The six amino acid differences in the external domains that distinguish 3DL1 from 3DS1 have been shown to be sufficient to abolish the interaction of 3DS1 with the HLA molecules containing the Bw4 epitope. Negative results from functional studies corroborate this lack of interaction, and indeed the only data implicating a function for 3DS1 are the widespread occurrence of the allele in all human populations combined with genetic association studies [19]. Genetic studies correlating *KIR3DS1* and *KIR3DL1* and *MHC-I* variation in HIV pathogenesis [20] led to the hypothesis that KIR3DS1 combined with the Bw4 epitope demonstrate genetic epistasis in controlling HIV infection [21]. However, no experimental evidence of a physical interaction between KIR3DS1 and HLA-Bw4 or any HLA class I allele grouping has been reported. It may then be worth investigating the possibility that instead, variable affinities between different allelic MHC-I OCs and KIR3DS1, possibly reflecting the Bw4 grouping, may account for that correlative relationship.

**Fig 5:**
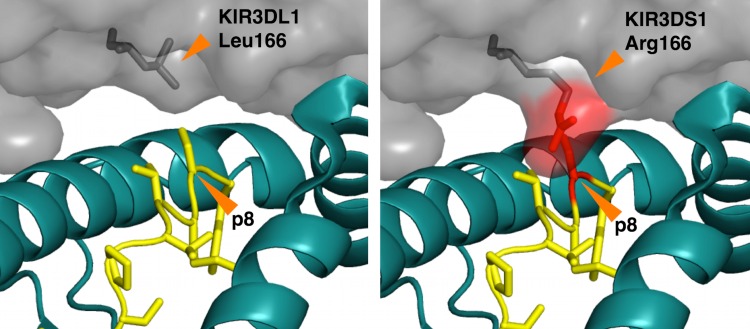
Steric clashes with presented peptides introduced by KIR substitutions. (*Left*) View of the KIR3DL1/HLA-B*5701 interface (PDB accession code 3VH8; www.rcsb.org). (*Right*) Model of KIR3DS1 in complex with HLA-B*5701, shown as at left. The steric clash between Arg166 with p8 is highlighted in red. One rotomer of Arg166 is depicted; however, all accessible Arg166 rotomers sterically clash with residue p8 of the bound peptide. HLA-B*5701 is shown in cartoon representation, colored teal; the bound peptide (LSSPVTKSF) is shown as a ribbon with side-chains in licorice stick representation, colored yellow. KIR3D molecules are shown in semi-transparent surface representation, colored grey. Residue 166 from the KIR3D molecules is shown in licorice stick representation, colored grey (KIR3DL1) or grey and red (KIR3DS1).

While no crystal structure of a KIR3DS1/HLA complex has yet been determined, a model of the complex between KIR3DS1*001 and HLA-B*5701 with bound peptide LSSPVTKSF has been made using the crystal structure of the complex KIR3DL1/ HLA-B*5701/ LSSPVTKSF [22]. This model shows that the Arg166 is the likeliest explanation for why 3DS1 does not bind intact peptide-MHC complexes, unlike 3DL1; the arginine side-chain clashes with the peptide in a hypothetical complex modeled on 3DL1 interactions supporting our finding that once MHC is without peptide, KIR3DS1 can bind (Fig 5). However, peptide-free MHC class I proteins–open conformers–likely adopt a significantly altered structure, distinct from an MHC class I protein structure simply without a peptide. Unfortunately, the open conformer structure is likely different enough that it is impossible to model in any meaningful way, given the paucity of structural data for this state. Therefore, while Arg166 may contribute to the lack of 3DS1 binding to MHC-I peptide complexes, we are not in a position to guess on structural details of the interaction between KIR3DS1 and open conformers or provide structural hypotheses for KIR3DS1 specificity and breadth.

The results reported in this study are consistent with a model previously presented that suggests that MHC-I expressed in the open conformer form, during an inflammatory response, act as ligands for activating KIR. The role of activating KIR may therefore be primarily during an inflammatory response when HLA-F and MHC-I OCs are upregulated [9]. During acute infection, this functional interaction may include communication between the innate and adaptive immune responses to further stimulate the adaptive response, as suggested by the role of HLA-F and MHC-I OC in a cross presentation pathway [10]. At another site of an inflammatory response, albeit of a different type, a role for HLA-C and KIR2DL1/2 in pregnancy has been postulated, with the lack of inhibitory signal between maternal KIR and placental HLA-C1 or C2 being correlated with better outcome [23]. In pregnancy, activation of decidual NK is likely necessary for secretion of cytokines and growth factors that enable vascularization essential for blood supply through the placenta [24]. The knowledge of HLA-F and other MHC-I open conformers as ligands for activating KIR and KIR3DL2, combined with their expression on placental and maternal cells respectively [25, 26], suggests that not only the suppression of inhibitory function but also activation through KIR may contribute to normal pregnancy.

During the review of this study, a complementary report suggesting HLA-F OC is a ligand for KIR3DS1 was published [27]. The respective studies differed on the point of whether HLA class I OCs bind to KIR3DS1, a conclusion in this study supported by SPR binding experiments (Fig 1 and S1 Fig). While the conversion of HLA complex to OC was supported by mAb and LILRB1/LILRB2 binding in SPR experiments (S2 Fig), no data was presented as control for conversion into OC in the complementary study where no binding was observed. Given that the experimental and technical approaches measuring interactions of HLA class I OC with KIR3DS1 differed between the studies, a definitive conclusion on this point remains the subject of further experimentation.

## Materials and Methods

### Protein Refolding and purification

The KIR2DS4 construct was tagged with an N-terminal HIS-tag for pull-down experiments and purified as previously described [11]. KIR3DL1 and KIR3DS1 cDNA encoding residues 1–314 (D0, D1, D2 domains and part of the stem region) were cloned with a C-terminus HIS-tag into the pRMH3 vector using EcoRI and BamHI restriction sites and expressed in Drosophila Schneider 2 (S2) cells (Invitrogen, Grand Island, NY) according to the manufacturer’s instructions. Both constructs contained a BIP leader (cleaved during expression). KIR recombinant proteins were purified by ion metal affinity chromatography using Ni-NTA resin (QIAGEN, Hilden, Germany) and then on a Superdex 200 10/300 GL (GE Healthcare, Pittsburgh, PA) liquid chromatography size exclusion column. KIR proteins were biochemically validated by analytical size exclusion chromatography, after final purification and concentration, showing monodispersivity (a single, clean peak at the correct molecular weight), and comparative reduced/non-reduced PAGE, showing purity and a single disulfide-linked species. Non-reduced PAGE is diagnostic for the correct folding of disulfide-linked proteins.

### Heterodimerization Assay

Refolded and purified HLA-F was incubated overnight at 4°C with KIR-His tagged proteins (2DS4, 3DS1, and 3DL1) in phosphate binding buffer containing 20 mM Imidazole. His-tagged proteins were captured on His SpinTrap columns (GE Healthcare, Pittsburgh, PA). After washing with binding buffer, protein complexes were eluted with phosphate buffer containing 500 mM imidazole.

### 3DS1 pull-down and Western blotting

Whole cells were incubated with purified 3DS1-His (and 3DL1-HIS) for one hour at 4°C, lysed, and proteins were captured on His SpinTrap columns (GE Healthcare, Pittsburgh, PA). Proteins were separated on 10% Bis-Tris gels (Invitrogen, Grand Island, NY) in reducing conditions and analyzed by Western blot using Abs 3D11 and HCA2 as described [8, 11].

### Surface plasmon resonance (SPR) analysis

Interaction between KIR3DL1/KIR3DS1 and HLA-F/MHCs was analyzed by SPR using a Biacore 3000 system at 25°C as previously described [8, 11]. Ligands (MHCs) obtained from the NIH Tetramer Core Facility (Emory University) were biotinylated through an engineered C-terminal BirA biotinylation site. Ligands were capture-immobilized (at 10 μl/min) on a SA sensor chip (approximately 1000 RU) immediately following repurification by size exclusion chromatography (SEC). Analytes were repurified by SEC in HBS-EP buffer within 48 h of use. Protein concentrations were determined using the BCA Protein assay (Pierce, Rockford, IL).

### PBMC stimulation

Frozen PBMC from de-identified healthy donors were thawed and resuspended in 10 mL RPMI-10% human serum overnight at 37°C. PBMC were stimulated 5:1 with irradiated 221 cells (3000 rad), at RPMI-10% human serum with 150 U/mL IL-2. T cells were activated with PMA (Sigma, St. Louis, MO) and ionomycin (Sigma) as described [9].

### CD107a assay

PBMCs were activated by IL-2 and 721.221 cells as described above and a CD107a assay was performed on day 8 post-stimulation. Activated PBMCs were incubated with 25μl of streptavidin microbeads, incubated previously for one hour with either HLA-F + β_2_m, D^b^-TRP- β_2_m or no protein, in the presence of anti-CD107a (clone H4A3, BD Pharmigen, Seattle, WA). GolgiStop (BD Biosciences, Seattle, WA) was added 1 h later according to manufacturer’s instruction. Cells were incubated for another 4.5 h then stained with LIVE/DEAD Fixable Violet Dead Cell stain, followed by anti-CD3-BV510 (clone OKT3, Biolegend, San Diego, CA), -CD56-PE (clone B159, BD Pharmigen), -KIR3DL1-FITC (clone DX9, BD Pharmigen), and -KIR3DL1/S1-APC (clone Z27, Beckman Coluter, Brea, CA). Finally, cells were fixed and washed with the BD Cytofix/Cytoperm kit. Flow cytometry and data analysis was performed as above.

## Supporting Information

S1 Fig KIR3DS1 binds to MHC-I open conformers. Binding sensorgrams of KIR3DL1 and KIR3DS1 as indicated above each graph to immobilized allelic MHC-I as indicated to the immediate left of each graph before (blue) and after (red) acid treatment. Experiments were performed in triplicates. KIR3DL1 and KIR3DS1 concentrations of 1 μM and 2 μM are indicated by light and dark colors respectively.
(TIF)

S2 Fig Controls for MHC surfaces. LILRB2 was used as a positive control for both free HCs and heterotrimeric MHCs. HCA2 and HC10 mAbs recognize free heavy chains and not MHC heterotrimeric complex and conformational antibody W6/32 make a clear distinction between heterotrimeric complex and free heavy chains, binding to complex but not to free HC (no β 2M, no peptide). Anti-β2M BB1M was used to monitor the absence after acid treatment.
(TIF)
