# Supplementary figures and images for: HLA-F and MHC-I Open Conformers Bind Natural Killer Cell Ig-Like Receptor KIR3DS1
Source: PLoS One. 2016 Sep 20;11(9):e0163297. doi: 10.1371/journal.pone.0163297 (PMC5029895; doi:10.1371/journal.pone.0163297)

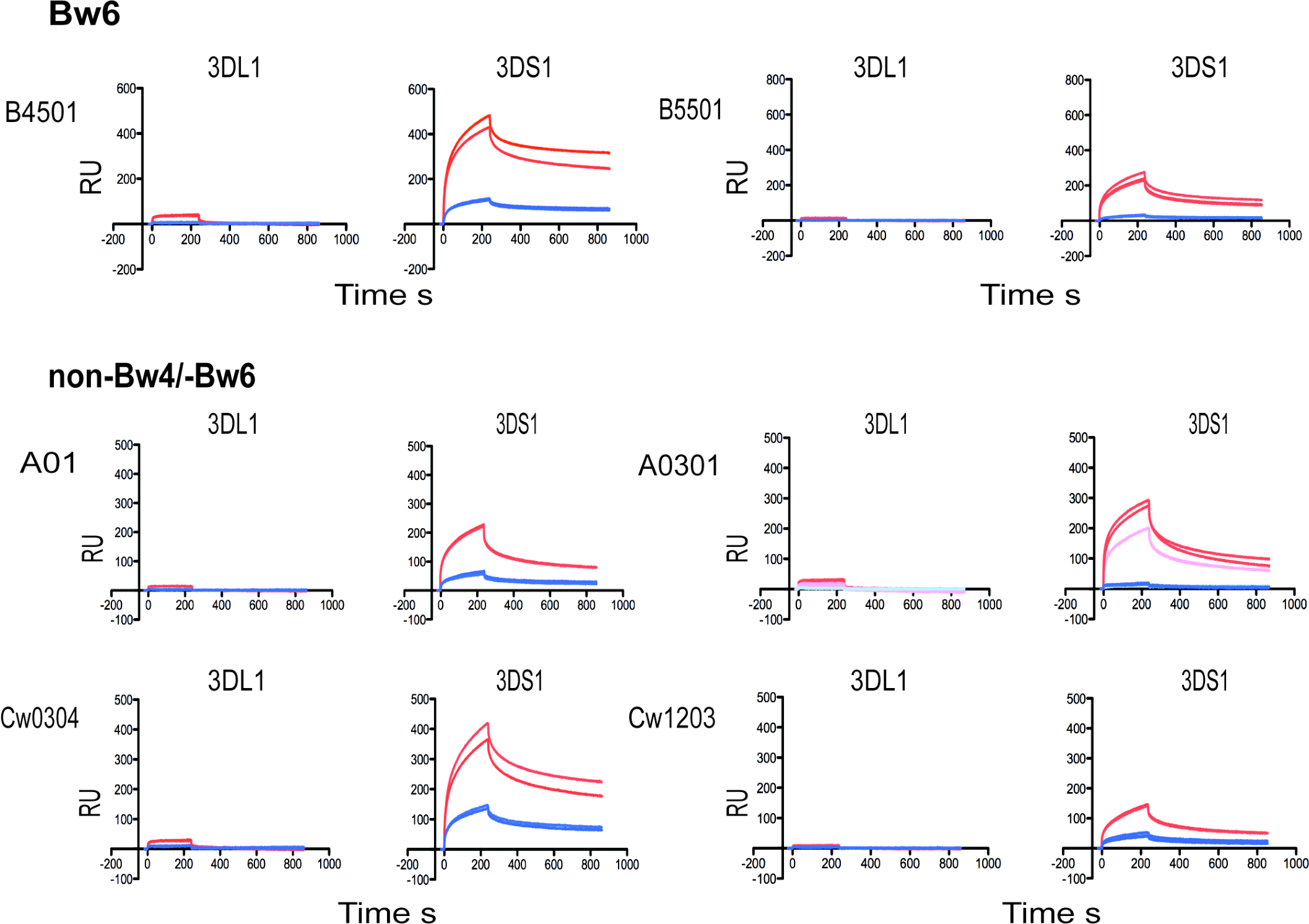

Supplement: S1 Fig — Binding sensorgrams of KIR3DL1 and KIR3DS1 as indicated above each graph to immobilized allelic MHC-I as indicated to the immediate left of each graph before (blue) and after (red) acid treatment. Experiments were performed in triplicates. KIR3DL1 and KIR3DS1 concentrations of 1 μM and 2 μM are indicated by light and dark colors respectively. (TIF) [file pone.0163297.s001.tif]

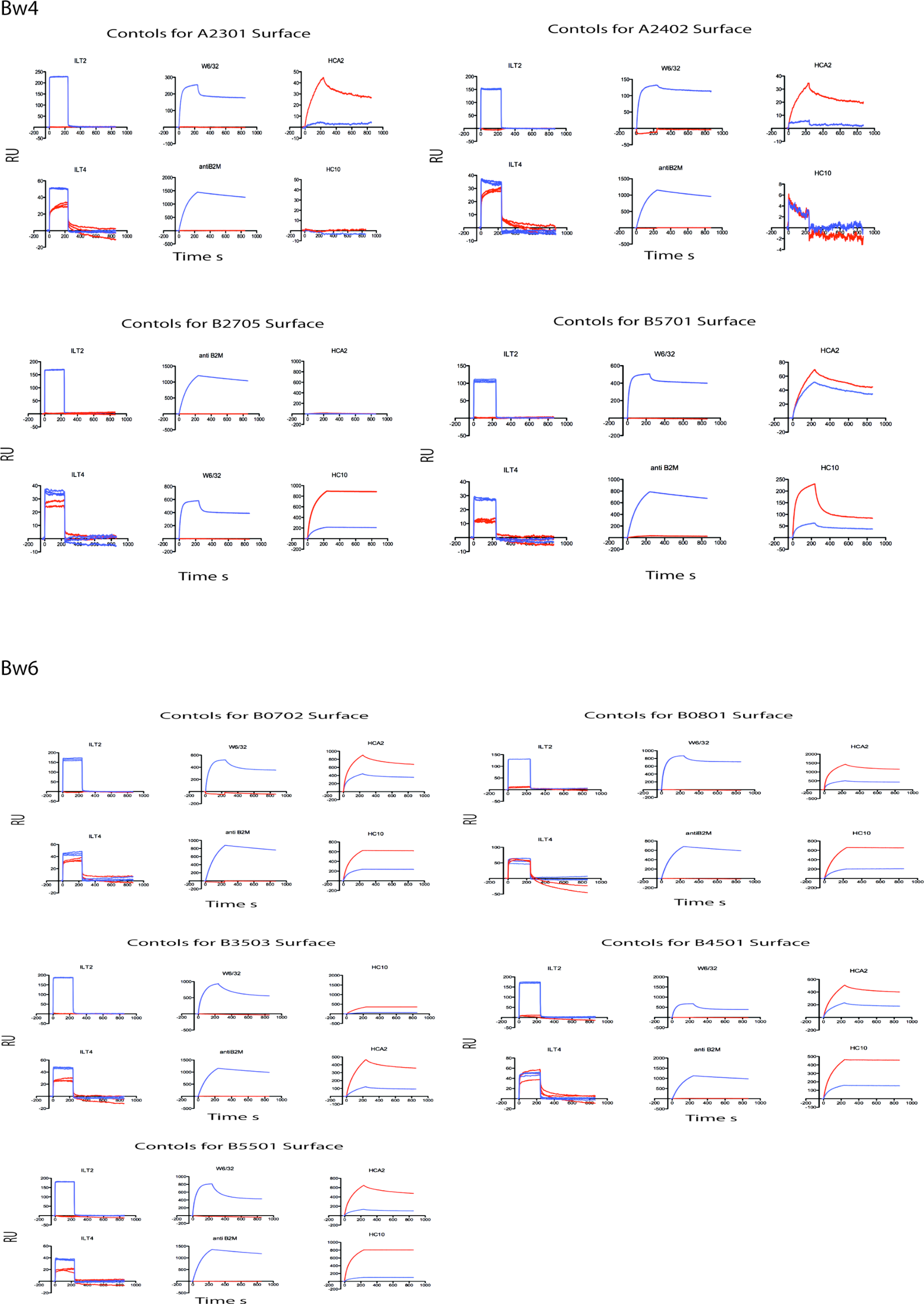

Supplement: S2 Fig — LILRB2 was used as a positive control for both free HCs and heterotrimeric MHCs. HCA2 and HC10 mAbs recognize free heavy chains and not MHC heterotrimeric complex and conformational antibody W6/32 make a clear distinction between heterotrimeric complex and free heavy chains, binding to complex but not to free HC (no β 2M, no peptide). Anti-β2M BB1M was used to monitor the absence after acid treatment. (TIF) [file pone.0163297.s002.tif]
